# Intrailiac Osteotomy With Superior Lateral Outcropping Bone: A Previously Undescribed Procedure for Hip Subluxation in Cerebral Palsy

**DOI:** 10.7759/cureus.42065

**Published:** 2023-07-18

**Authors:** Benjamin Sketchler, David A Yngve

**Affiliations:** 1 Orthopaedic Surgery and Rehabilitation, University of Texas Medical Branch, Galveston, USA

**Keywords:** hip subluxation, pelvis, osteotomy, congenital hip dysplasia, cerebral palsy (cp)

## Abstract

Background: Surgical treatment of hip subluxation in cerebral palsy typically involves proximal femoral osteotomy with or without concurrent supra-acetabular pelvic osteotomy. The literature lacks data on isolated pelvic osteotomy for this condition. We present superior lateral outcropping bone as a novel procedure for augmenting pelvic osteotomies for additional femoral coverage.

Methods: In this retrospective case series, all patients were included for whom a single surgeon at a single institution performed pelvic osteotomy with adjunctive superior lateral outcropping bone for the treatment of hip subluxation in cerebral palsy over a 12-year period. Patients with less than two years of X-ray follow-up were excluded, as were patients with frank dislocation preoperatively. Regarding each case, multiple variables were collected, including X-ray measurements of migration percentage and acetabular index preoperatively, immediately postoperatively, and at last available X-ray. Paired *t* tests were performed to confirm a significant difference between preoperative and postoperative measurements. Surgical failure was defined as either any subsequent hip or pelvic procedure other than myotendinous lengthening or alcohol nerve blocks, or final migration percentage of greater than 50%.

Results: Thirty-three hips (23 patients, 13 males) were included. Mean age at surgery was seven years. Mean time to follow-up was 49 months. Migration percentage of the hips improved from an average 44% preoperatively to 25% at first postoperative measurement and 22% at final follow-up (p < 0.001). Acetabular index improved from an average 27 degrees preoperatively to 15 degrees at first postoperative measurement and 17 degrees at final follow-up (p < 0.001). No hips met failure criteria of repeat surgery other than myotendinous lengthening or nerve blocks, but two presented with a migration percentage of greater than 50% at final follow-up, giving us a failure rate of 6%.

Conclusions: We present a novel procedure that appears to provide safe and successful outcomes for hip subluxation in cerebral palsy. Our clinical results compare favorably to those in the literature for isolated proximal femoral osteotomy for similar patient populations, yet there is no need for implanted hardware.

## Introduction

Painful hip dislocation commonly occurs in nonambulatory children [1]. Surgical treatment of hip instability in cerebral palsy has evolved over the years. As the outlook on this condition has evolved, so have treatment options.

Iliac or femoral osteotomy for developmental dysplasia of the hip

The treatment of hip dysplasia from cerebral palsy is a distinct topic from the treatment of hip dysplasia from developmental dysplasia of the hip (DDH) because of the neuromuscular component in those with cerebral palsy. However, it can be helpful to mention two prominent articles on the use of iliac osteotomies in the treatment of DDH from the 1960s because, at the time, osseous hip reconstruction was more common in DDH than in cerebral palsy. The articles are single-author studies by Pemberton and by Salter describing the iliac osteotomies later known by their names [2-3]. Following this early interest in isolated iliac osteotomy was interest in treating DDH with isolated femoral osteotomy. A difficulty with high dislocations was bringing the femoral head down to the level of the acetabulum. A common option was pre-surgery traction. Another means was femoral shortening osteotomy, which gained popularity. One report advocated open reduction and femoral shortening osteotomy in all hips [4]. Another study maintained that open reduction with femoral osteotomy had lower rates of partial avascular necrosis than open reduction with pelvic osteotomy [5]. A further development was combined iliac and femoral osteotomy for DDH [6-7].

Combined iliac and femoral osteotomy in cerebral palsy

Often, combined iliac and proximal femoral osteotomy are used to treat hips with dislocation or significant subluxation in those with cerebral palsy. At some centers, it is standard to combine both osteotomies [8]. At other centers many, but not all, patients were reported to have both osteotomies [9-12].

Isolated femoral osteotomy for cerebral palsy

For hips that have subluxation but not dislocation, isolated proximal femoral osteotomy is often chosen [9]. Isolated femoral osteotomy is effective in decreasing the uncovered percentage, but not the acetabular index. In one study of children under the age of six at surgery who had greater than four years of follow-up, uncovered percentage improved from 52% to 24%, but acetabular index improved only from 25 degrees to 23 degrees [13]. In another study, uncovered percentage improved from 51% to 26%, but acetabular index improved only from 28 degrees to 25 degrees [9].

Isolated femoral osteotomy for unilateral dysplasia can lead to dysplasia requiring bony surgery on the contralateral side. In one study of unilateral femoral osteotomies, 44% required operation on the other side at a mean follow-up of five years. The authors surmised that unilateral surgery causes force changes that lead to pelvic obliquity. That obliquity then puts the opposite side at risk [14]. Another study reported a 37% failure rate in 320 patients after femoral osteotomy when followed eight years [15].

Decision making - one vs. two osteotomies in cerebral palsy

Algorithms have been created to determine if a given hip should be treated with a femoral osteotomy alone, or with combined femoral and iliac osteotomies [9]. There is evidence that isolated femoral osteotomy cases may be likely to fail if, preoperatively, the femoral head is uncovered greater than 48% [16].

Isolated iliac osteotomy with cerebral palsy

Although isolated iliac osteotomy was described in DDH by Salter and Pemberton, the situation is different for the hip in cerebral palsy. To our knowledge, there is no dedicated paper on the treatment of hip dysplasia in cerebral palsy with isolated iliac osteotomy, because in all reported series of pelvic osteotomy for hip dysplasia in cerebral palsy, a femoral osteotomy was combined with the iliac osteotomy for most, if not all, of the cases [8, 10-12].

In a systematic review of 36 articles on osteotomies for hip dysplasia in cerebral palsy, combined femoral and pelvic osteotomies were performed in 53% (1374 hips), femoral osteotomy only in 46% (1173 hips), and pelvis osteotomy only in 0.8% (21 hips). Those 21 hips came from three studies that used isolated iliac osteotomy only at times [17].

The use of isolated proximal femoral osteotomy for the treatment of hip dysplasia in cerebral palsy has several difficulties. It improves uncovered percentage, but not acetabular index. Unilateral surgery has a high percentage of deterioration of the other side by altering hip forces. Hardware removal adds an additional surgery.

There is a need for a study of iliac osteotomy for the treatment of hip dysplasia in cerebral palsy. This study should be of iliac osteotomy only without the use of femoral osteotomy. It should have enough patients and adequate follow-up so that the procedure can be adequately evaluated. Our purpose is to present such a study.

Further, our study provides a description of a novel procedure: superior lateral outcropping bone. This is performed as an adjunct to the iliac osteotomy in our patients. It involves augmenting the redirected acetabulum with a combined allograft-autograft mixture for additional femoral coverage. Our goal is to demonstrate that these combined procedures provide a reasonable alternative to isolated femoral osteotomy or to combined femoral and iliac osteotomies in the setting of hip subluxation in cerebral palsy.

## Materials and methods

This study received approval from the University of Texas Medical Branch Institutional Review Board (study approval #07-166), citing "Written documentation of consent is waived in accordance with 45 CFR 46.117(c)." Our study is a retrospective case series with a quantitative approach. Institutional Review Board approval and appropriate consent were obtained. This was a single-surgeon study of intrailiac osteotomy with superior lateral outcropping bone for hip subluxation in cerebral palsy, from the years 2007 to 2019. Patients who also had same-setting ipsilateral femoral osteotomy were not included in the study. Forty-four hips (33 patients) fit these criteria. Exclusion criteria included a follow-up period of less than two years, for which nine of these 44 hips were excluded. Two cases were excluded due to preoperative migration percentages that exceeded our proposed indication of 40%-50%.

**Figure 1:**
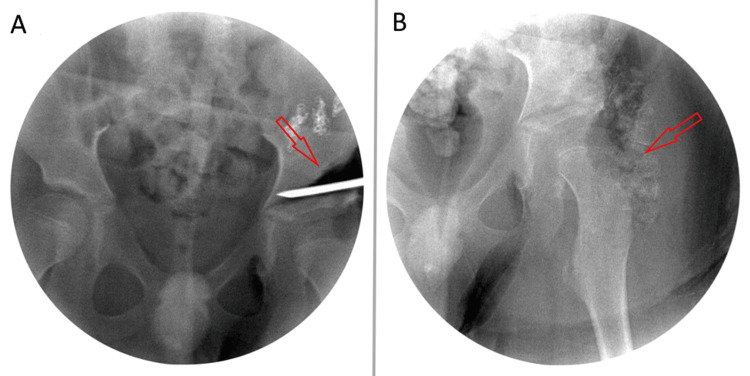
Intraoperative fluoroscopy. A: Performing the iliac osteotomy. B: After osteotomy and application of superior lateral outcropping bone graft.

The surgical technique involves exposure of the hip capsule and iliac wing via a modified Smith-Petersen approach. A sartorius release, rectus femoris release, and psoas lengthening are performed. A supra-acetabular osteotomy is performed with osteotomes as described by Dega, using fluoroscopy, with wedges of iliac crest bone used to maintain the new acetabular positioning [12]. We then construct the superior lateral outcropping bone by packing a mix of autogenous bone marrow aspirate, cancellous allograft, and demineralized bone matrix adjacent to the outer table of the ilium. This forms an outcropping from the ilium above the acetabular roof that extends laterally and distally past the superior margin of the femoral head. Typically, we might mix bone cubes of 2 mL/kg each side with demineralized bone matrix gel 1 mL/kg each side with bone marrow aspirate 10 ml for each side. For a 20 kg child, this would be cancellous allograft cubes 40 mL with demineralized bone matrix gel 20 mL and bone marrow aspirate 10 mL for each side. Then we would place the amount that comfortably fits. Because the hip subluxation is commonly in a posterior as well a superior direction, it is important to dissect posteriorly along the ilium so that the bone graft will sit laterally and posterolaterally. Also, if the graft is too anterior it could impair hip flexion. The dissection continues from the ilium distally over the hip capsule, which is left intact. A pouch is created extending laterally from the ilium and the hip capsule, and this can be held open with a soup spoon. The spoon is bent at about 60 degrees where the handle attaches to the spoon. The spoon is then placed into the posterior depths of the pocket to hold open the space for the graft (Figure 1).

**Figure 2:**
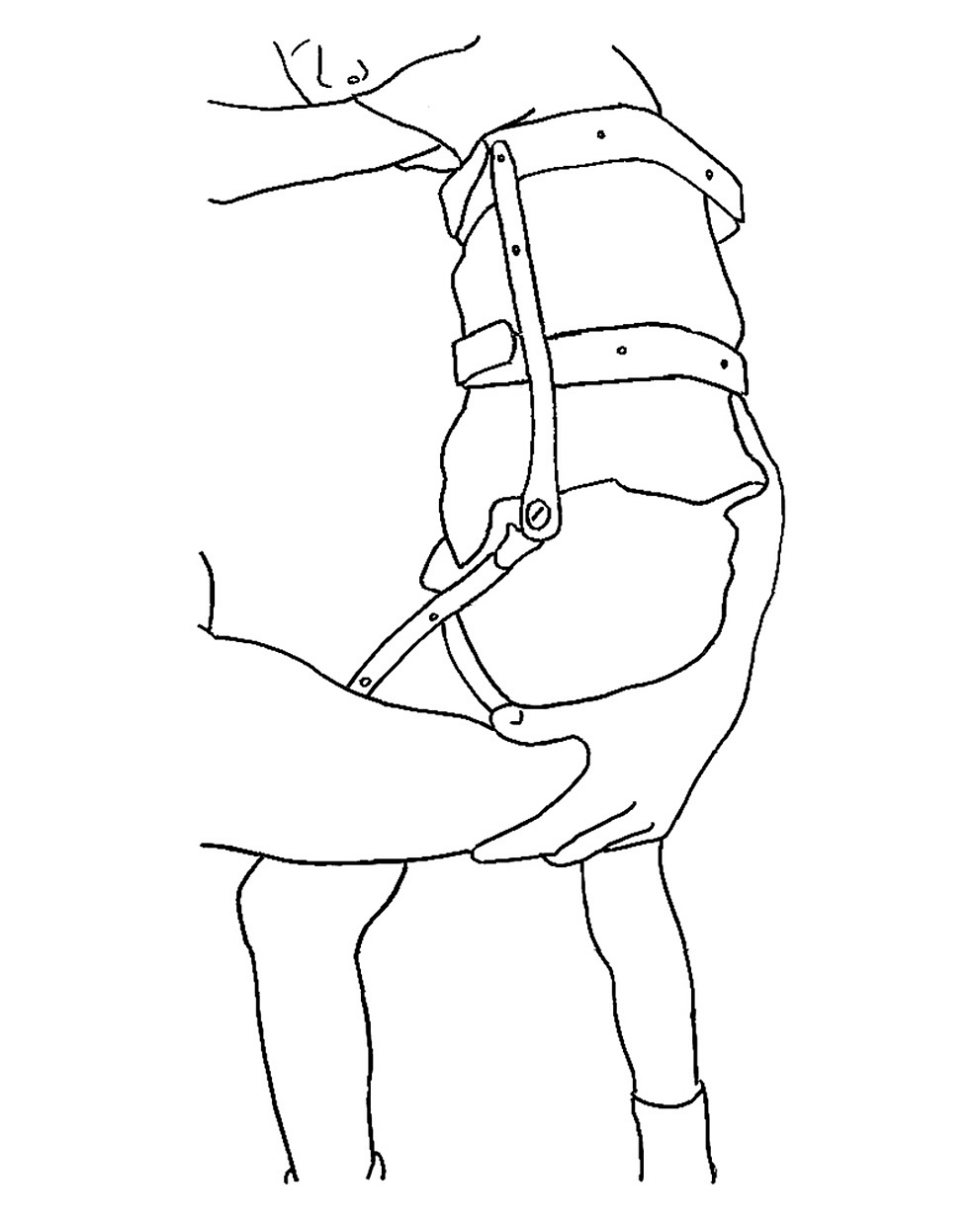
A hip orthosis was worn for 3-6 months following the procedure. The upper extent of the orthosis should be two finger widths below the axillae for proper control. The abduction is adjusted so that the outside shoulder width would fit between the knees. This figure was adapted from an unpublished photograph taken by Roy M. Nuzzo, MD and given to the senior author with full permission to use and reproduce.

The wound is closed in standard fashion. Hip abduction bracing was used nearly full-time for three to six months with an orthosis that extended from below the axillae to the thighs, with free hip hinges. The hips were equally abducted so that the there was a shoulder-width distance between the knees (Figure 2). The operative technique, postoperative care and indications were based on the teachings and descriptions of Roy Nuzzo, MD [18].

Variables collected on each case included demographics, preoperative Gross Motor Function Classification System (GMFCS) levels, and other surgeries performed concurrently with the index procedure. For each case, anteroposterior X-rays were examined preoperatively, in the immediate postoperative period, and at latest follow-up. Values for the femoral head uncovered percentage and the acetabular index were recorded by a single researcher. X-rays from first clinic follow-up were used for the immediate postoperative time point, with patients in their hip abduction braces, unless the first available X-ray was obtained more than six weeks postoperatively, in which case an immediate postoperative X-ray under anesthesia and in their brace was utilized instead. Frequency analysis was performed on these X-ray values for all three time points, and paired t tests were performed to determine whether there was a significant difference between preoperative and final postoperative values for migration and acetabular index.

Charts for all patients were reviewed through the end of the data collection period for repeat surgery on each operated hip. For these repeat surgeries, a note was made whether these included only soft-tissue releases and nerve blocks or if any had more extensive procedures, such as irrigation and debridement or subsequent femoral osteotomy. Surgical failure was defined as any subsequent hip or pelvic procedure other than musculotendinous lengthening or nerve block. A final migration percentage of greater than 50% was also considered a surgical failure, to parallel a value used elsewhere in the literature [15].

## Results

Our study included 33 hips (23 patients, 13 males; Table 1). Mean age at surgery was seven years (range, 3-16 years). Mean time from surgery to final follow-up X ray was 49 months (standard deviation, SD 16 months, maximum time 109 months). Preoperative GMFCS levels ranged from two (one patient) to five (median, 4). Of 33 hips included in our study, 20 were reconstructed bilaterally.

**Table 1:** Demographics. GMFCS, Gross Motor Function Classification System; SD, standard deviation

Sex	Number
Male	18
Female	15
GMFCS level (no. [%])	
2	1 (3%)
3	8 (24%)
4	12 (36%)
5	12 (36%)
Age (years +/- SD)	7 +/- 3
Bilateral surgery (no. [%])	20 (61%)
Duration of follow-up (mo +/- SD)	49 +/- 16

Concomitant procedures are shown in Table 2. Twenty-three had concomitant percutaneous adductor tenotomy (seven bilateral, 13 only ipsilateral, and three only contralateral). All had concomitant alcohol obturator nerve block (17 bilateral, 13 only ipsilateral, and three only contralateral). Four had concomitant percutaneous hamstring lengthening at the knee (three bilateral and one only ipsilateral). Eleven had percutaneous gastrocnemius-soleus complex lengthening (nine bilateral and two only ipsilateral). Other concomitant procedures included other musculotendinous lengthening (Achilles, peroneal, sartorius, gracilis, biceps brachii), other alcohol nerve blocks (biceps brachii, pectoralis), and in one case, a subtalar arthrodesis and great toe flexor tenotomy.

**Table 2:** Concomitant procedures.

Obturator nerve block	29
Adductor release	23
Gastrocnemius release	13
Hamstring release	4
Pectoralis nerve block	3
Distal femoral osteotomy - open	2
Subtalar arthrodesis - open	2
Toe flexor release	2
Achilles tenotomy	1
Peroneal release	1
Biceps brachii release	1

**Figure 3:**
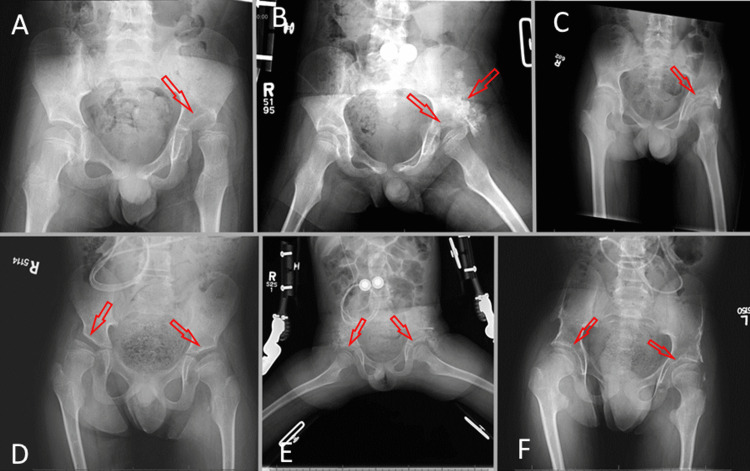
Sample radiographs. A: Patient 1, preoperative. B: Patient 1, first postoperative X-ray, left-side surgery. C: Patient 1, final postoperative X-ray. D: Patient 2, preoperative. E: Patient 2, first postoperative X-ray, bilateral surgery. F: Patient 2, final postoperative X-ray. Note: Some patients have more prominent superior lateral outcropping bone at final follow-up than others. We have not detected an association between outcomes and X-ray persistence of superior lateral bone.

On frequency analysis of X-ray findings, the mean preoperative migration percentage was 44% (Table 3). This value improved to 25% at first postoperative X-ray and remained improved at 22% at final follow-up. The mean preoperative acetabular index was 27 degrees. This value improved to 15 degrees at first postoperative X-ray and remained improved at 17 degrees at final follow-up (Figure 3).

**Table 3:** Frequency analysis. SD, standard deviation

	Uncovered percentage			Acetabular index		
	Mean	SD	Median	Mean	SD	Median
Preoperative	44	8	50	27	5	28
First postoperative	25	12	25	15	6	16
Final	22	17	20	17	10	17

Paired *t* tests were performed comparing the preoperative and final postoperative values both for migration percentage and acetabular index, both demonstrating a p value of less than 0.001 for significance of difference between groups. Of the 33 hips, 14 had repeat surgery by the end of the data collection period. However, these repeat surgeries were all limited to musculotendinous lengthening, typically solely adductor tenotomy, typically with obturator neurolysis. No repeat surgeries were required for infection, further osteotomies, or other reasons. No hips met failure criteria of repeat surgery other than myotendinous lengthening, but two presented with a migration percentage of greater than 50% at final follow-up, giving us a failure rate of 6%.

## Discussion

Isolated iliac osteotomy with superior lateral outcropping bone provides safe and successful outcomes for hip subluxation in cerebral palsy. This study adds to the minimal literature regarding isolated pelvic osteotomy for the treatment of hip subluxation in cerebral palsy [17]. We acknowledge that proximal femoral osteotomy provides clinical value in the right setting. We perform combined femoral and pelvic osteotomies routinely in patients with cerebral palsy with migration percentages greater than 50%. However, surgeons interested in avoiding the need for metal implants or interested in forgoing other difficulties inherent with femoral osteotomy may find value in this procedure. Further, our data demonstrate significant lasting improvement in the acetabular index; contrarily, isolated femoral osteotomies do not appear to be associated with significant acetabular remodeling (Table 4) [13, 19].

**Table 4:** Iliac osteotomy vs. femoral osteotomy.

	Iliac osteotomy	Femoral osteotomy
Significantly improves migration percentage	Yes	Yes
Significantly improves acetabular index	Yes	No
Avoids leg shortening	Yes	No
Avoids major length changes to muscles that cross the hip joint	Yes	No
Avoids the use of hardware	Yes	No
Avoids possible hardware removal	Yes	No

There seems to be a basic difference in the biologic response between isolated femoral osteotomy and isolated iliac osteotomy. In this study, there was marked improvement in both the migration percentage, from 44% to 25%, and the acetabular index, from 27 to 15 degrees. In contrast, in reports of isolated femoral osteotomy, there was also marked improvement in migration percentage, from 51% to 26% and from 52% to 24% in two studies [9, 13]. However, those studies showed only very little change in the acetabular index, from 28 degrees to 25 degrees and from 25 degrees to 23 degrees. The migration percentage and the acetabular index are both important and widely used measures of hip health. Our study demonstrated marked improvement in both measures, whereas studies of isolated femoral osteotomy show marked improvement in only one measure. 

Our study has limitations. It is a nonrandomized case series without in-study control. Also, there is no comparison group of pelvic osteotomies without adjunctive superior lateral outcropping bone. Although many studies describe results of bony hip reconstruction in cerebral palsy, many different outcome measures are used, radiographic and otherwise, over varied time points [1, 8, 15, 17]. Similarly varied results exist for interesting new treatment concepts, such as proximal femur guided growth [20-21].

Despite this difficulty in comparison, our results do compare favorably to those in other studies. In a recent cohort of 567 proximal femoral osteotomies with or without concomitant pelvic osteotomy for children with cerebral palsy, 9% of hips had osseous revision surgery within five years, compared with our zero osseous revisions and 6% failure after a mean 49 months [15]. In another recent cohort of 199 hips with cerebral palsy treated with isolated proximal femoral osteotomy, 25% of hips demonstrated migration percentage greater than 33% at final follow-up after a mean of 5.4 years, compared to 18% of our hips [22].

Other limitations should be mentioned. Our series relies primarily on X-ray outcomes. The clinical data available precluded effective measurement of other outcomes. Also, questions remain as to the generalizability of this procedure, performed here by one surgeon at a single institution.

## Conclusions

This is a case series of 33 subluxated hips in 23 patients with cerebral palsy, all treated with supra-acetabular iliac osteotomy with adjunctive superior lateral outcropping bone. Significant improvement was seen in X-ray outcomes. The results are competitive with those of other procedures, with a clinical failure rate of only 6% for a condition that can be difficult to treat. Our data support that this novel procedure for the treatment of hip subluxation in cerebral palsy may serve as a valid alternative to other described surgical options. Further study is needed.
